# Examining Structural Disparities in US Nursing Homes: National Survey of Health Information Technology Maturity

**DOI:** 10.2196/37482

**Published:** 2022-08-23

**Authors:** Gregory L Alexander, Jianfang Liu, Kimberly R Powell, Patricia W Stone

**Affiliations:** 1 School of Nursing Columbia University New York, NY United States; 2 Sinclair School of Nursing University of Missouri Jefferson City, MO United States

**Keywords:** nursing homes, health information technology, policy, nursing informatics, electronic health record, electronic data, data sharing, care providers, resident, care, quality of care, structural disparity, clinical support, administration

## Abstract

**Background:**

There are 15,632 nursing homes (NHs) in the United States. NHs continue to receive significant policy attention due to high costs and poor outcomes of care. One strategy for improving NH care is use of health information technology (HIT). A central concept of this study is HIT maturity, which is used to identify adoption trends in HIT capabilities, use and integration within resident care, clinical support, and administrative activities. This concept is guided by the Nolan stage theory, which postulates that a system such as HIT moves through a series of measurable stages. HIT maturity is an important component of the rapidly changing NH landscape, which is being affected by policies generated to protect residents, in part because of the pandemic.

**Objective:**

The aim of this study is to identify structural disparities in NH HIT maturity and see if it is moderated by commonly used organizational characteristics.

**Methods:**

NHs (n=6123, >20%) were randomly recruited from each state using Nursing Home Compare data. Investigators used a validated HIT maturity survey with 9 subscales including HIT capabilities, extent of HIT use, and degree of HIT integration in resident care, clinical support, and administrative activities. Each subscale had a possible HIT maturity score of 0-100. Total HIT maturity, with a possible score of 0-900, was calculated using the 9 subscales (3 x 3 matrix). Total HIT maturity scores equate 1 of 7 HIT maturity stages (stages 0-6) for each facility. Dependent variables included HIT maturity scores. We included 5 independent variables (ie, ownership, chain status, location, number of beds, and occupancy rates). Unadjusted and adjusted cumulative odds ratios were calculated using regression models.

**Results:**

Our sample (n=719) had a larger proportion of smaller facilities and a smaller proportion of larger facilities than the national nursing home population. Integrated clinical support technology had the lowest HIT maturity score compared to resident care HIT capabilities. The majority (n=486, 60.7%) of NHs report stage 3 or lower with limited capabilities to communicate about care delivery outside their facility. Larger NHs in metropolitan areas had higher odds of HIT maturity. The number of certified beds and NH location were significantly associated with HIT maturity stage while ownership, chain status, and occupancy rate were not.

**Conclusions:**

NH structural disparities were recognized through differences in HIT maturity stage. Structural disparities in this sample appear most evident in HIT maturity, measuring integration of clinical support technologies for laboratory, pharmacy, and radiology services. Ongoing assessments of NH structural disparities is crucial given 1.35 million Americans receive care in these facilities annually. Leaders must be willing to promote equal opportunities across the spectrum of health care services to incentivize and enhance HIT adoption to balance structural disparities and improve resident outcomes.

## Introduction

### Background

There are 15,632 nursing homes (NHs) in the United States with 1.7 million beds and 1.3 million Americans residing in them [1]. For decades, care delivered in NHs has received significant policy attention due to the poor outcomes of care [2]. Recently, national experts in NH care were charged with examining how our nation delivers, regulates, finances, and measures quality [3]. The committee concluded that the current state of quality of care in NHs is “…ineffective, inefficient, fragmented, and unsustainable” [4]. NH quality has several components that interact to affect residents’ health, functional status, and resident outcomes. Researchers have studied these interactions to understand how policy (ie, regulation and reimbursement), clinical interventions, management practices, and individual worker, resident, and family characteristics account for variation in NH quality [5]. A promising strategy for improving NH quality is the use of health information technology (HIT).

In this paper, we define HIT as a system that is used in health care to process, store, and exchange health information in an electronic environment. The use of HIT in NHs has also been recognized by experts in the field as a method to improve NH quality [6]. For example, NH HIT improves timely and secure exchange of electronic data and medical record access, enabling clinicians to gain direct access to clinical information, enhances efficiency, and improves resident outcomes [7,8]. Furthermore, HIT strengthens care coordination processes leading to greater consistency and better accountability among clinicians and staff [9]. Unfortunately, wide differences in NH HIT adoption exist. Researchers have found that 95% of NHs use HIT including electronic health records (EHR), and nearly half (46%) use health information exchange capabilities for resident care. However, they also discovered variations in use of technology, such as an EHR (ie, urban NHs were 2.5 times more likely to have EHR compared to rural NHs) [10]. One element missing from national NH quality reporting systems includes measures of the maturity of HIT adoption, which could help policy makers, researchers, quality improvement specialists, families and residents, and other stakeholders to identify where gaps exist.

### Theoretical Approach

A central concept of this study is HIT maturity guided by stage theory by Nolan [11], which postulates that a system of coordinated processes (eg, an EHR) evolves through a series of stages as it matures. HIT maturity models, such as the Health Information Management System Society Electronic Medical Record Adoption Model, are used to assess levels of EHR maturity over time in acute care, and other maturity models are used to assess the general health care environment, mobile health, interoperability, telemedicine, and usability [12-14]. These general models of HIT maturity are not adaptable to NH contexts because NHs provide a different model of care delivery [15]. For instance, NH residents’ length of stay is typically much longer than that of a patient in acute care. Furthermore, providers are largely off-site, and the nursing workforce is different in NHs compared with other settings (eg, NHs have higher staffing of licensed practical nurses, and the bulk of the workforce are certified nursing aides) [16]. Other HIT maturity models have been proposed that stress the importance of continuous cycles of reassessment that could be influenced by policy or change in solutions [17]. Previously, we have defined NH HIT maturity in 3 dimensions including HIT capabilities, use, and integration; these dimensions are further defined among the following 3 health care domains: resident care, clinical support (eg, laboratory, pharmacy, and radiology), and administrative activities. Furthermore, NH HIT maturity is categorized into 7 stages ranging from stage 0 “nonexistent HIT or EHR solutions” to stage 6 “integrated HIT systems that generate clinical data to drive self-management” [11]. HIT maturity is best measured longitudinally to enable better estimates of change in adoption over time [18]. Widespread NH HIT maturity has not been achieved due to unbalanced national policies, which have not promoted meaningful use or provided financial incentives in NHs for HIT adoption, similar to other health care sectors [19,20]. This imbalance has created wider structural disparities leading to variation in resource capabilities and use that may influence disparities in resident outcomes. For instance, following the emergence of the COVID-19 pandemic in March 2020, early analyses of telehealth use found that compared to rural NHs, urban NHs were more than 11 times more likely to use telehealth for web-based evaluations, pretransfer arrangements, second opinions, and transfer of diagnostic images following major policy changes [21,22]. Ongoing NH assessments are critical to truly understand the linkages between these types of organizational differences and impacts on quality of NH care, including disparities in resident outcomes.

HIT maturity is an important component of the rapidly changing NH landscape. In previous work, we have found alternating patterns of total HIT maturity over 3 years (2014-2017) among 815 NHs; that is, (n=579, 71%) of NHs exhibited net positive increase in total HIT maturity, (n=155, 19%) had a net negative decrease in total HIT maturity, and (n=58, 10%) had consistently negative patterns of total HIT maturity over time [18,23]. Facilities with a net increase reported increasing HIT capabilities and use as well as greater integration over time. However, NHs, reporting a net decrease in HIT maturity over time, have reduced their capabilities, use, and system integration. For instance, one of the areas that had the most variation over time in HIT maturity was the clinical support dimension associated with laboratory, pharmacy, and radiology technologies used in NHs [23]. Clinically, this makes sense, since NHs that are unaffiliated with hospitals typically do not have a laboratory, pharmacy, or radiology department in house, so they will oftentimes use disparate, stand-alone technologies to facilitate related activities for staff and residents. These disparate, isolated systems may be easy to remove if they are not found to be efficient, are too costly, or do not meet the expectations of the users.

The development of HIT maturity surveys has allowed researchers to begin exploring the relationship between technology use and NH resident level outcomes. For instance, recent studies have revealed mixed associations between HIT maturity and antibiotic use and urinary tract infections. In one study, linking HIT maturity data with a resident-level minimum data set yielded 219,461 regular resident assessments within 90 days of survey completion on 80,237 unique, older adult long-stay residents. We found that for every 10-point increase in the HIT maturity score, the expected odds of antibiotic use increased by 7% [24]. Although this result was unexpected, NHs with higher HIT maturity may have enhanced systems enabling nurses to monitor when antibiotics are used; therefore, higher levels of awareness could eventually lead to reductions in inappropriate antibiotic use. Additionally, we examined associations between HIT maturity and urinary tract infections. Controlling for NH and resident characteristics, HIT maturity was associated with 10% less urinary tract infections [25].

The COVID-19 pandemic has also influenced changes in HIT use [22,26]. These changes include the expansion of Medicare payment for telehealth, increasing collaborations between academic medical centers and NHs, and the Centers for Medicare & Medicaid Services requiring NHs to report COVID-19 metrics to the Centers for Disease Control electronically [21,27,28]. Following national telehealth policy expansion in Spring 2020, the use of NH telehealth using computer software and web applications surged across the country [22]. However, 16% of NHs were not using telehealth, and this was more likely to occur in rural NHs [22]. Similar findings have been confirmed by other researchers exploring the explosive growth of telehealth since the pandemic started [29]. Technology adoption, especially in the face of emergent conditions, yields positive and negative outcomes that must be recognized, identified, and addressed [10]. The purpose of this study was to identify structural disparities in NH HIT maturity and how HIT maturity is moderated by various commonly used NH characteristics (eg, ownership, location, number of certified beds, chain affiliation, and occupancy rate).

## Methods

### Ethics Approval

This research was conducted as part of a larger ongoing 3-year national study (2019-2022) exploring trends in NH HIT maturity in the United States. Data were collected in 2019 using an NH survey that measures 3 dimensions of HIT maturity (ie, HIT capabilities, use, and integration) in the 3 domains of resident care, clinical support (ie, laboratory, pharmacy, and radiology), and administrative activities. All methods used in this research were approved by the Columbia University Institutional Review Board (PT-AABR3810).

### Sample

Nursing home compare is a publicly available database, maintained by the Centers for Medicare & Medicaid Services, which provides information about every US NH serving beneficiaries of Medicare or Medicaid [1]. Nursing home compare was used to identify NHs in continental US, Alaska, and Hawaii. NHs were excluded from Guam, Puerto Rico, and Virgin Islands. NHs designated as a *special focus facility* were also excluded, because these facilities have a history of serious quality issues and are automatically included in a program to stimulate quality-of-care improvements [30]. Finally, NHs with a hospital-based designation was not included as their HIT maturity is likely different due to national incentives for HIT adoption in acute care [8,31]. After applying the exclusion criteria, the population size was 14,109 (Table 1).

The sample recruitment goal for this study was 10% of all NHs in the United States (N=1570 NH). Based on our previous experience with a 45% response rate, more than 20% (n=6123) of the facilities were randomly recruited from each state. The number of facilities selected in each state was proportional to the number of NHs located in that state. For example, because California has the largest number of homes (n=1241), 248 homes (20% of facilities) were randomly selected from all California NHs. To ensure that responses were received from multiple NHs in each state, we overrecruited in states with smaller numbers to have a minimum of 6 homes per state. Although sampling was stratified by state, facilities were not stratified further by the number of certified beds, ownership, location, and so on prior to recruitment; this is because some states may not have any NHs in some strata. Wyoming, for example, has only 38 homes. In Wyoming, there are fewer large homes in rural areas. By including every NH in the random selection process within each state, every home in each state—regardless of their characteristics—had an equal opportunity to participate.

**Table 1:** Comparison of national nursing home population vs sample.

Nursing home characteristics			National (N=14,109)		Sample (n=719)		Probability ratio or Cohen *d*^a^		*P* value	
**Location, n (%)**										.07
	Metro >50,000	9823 (69.68)		453 (63)		1.11				
	Micro 10,000-49,999	1936 (13.73)		114 (15.86)		0.87				
	Small town 2500-9999	1414 (10.03)		90 (12.52)		0.80				
	Rural <2500	925 (6.56)		62 (8.62)		0.76				
**Number of certified beds, n (%)**										<.001
	<60	2418 (17.14)		150 (20.86)		0.82				
	60-120	7582 (53.74)		420 (58.41)		0.92				
	>120	4109 (29.12)		149 (20.72)		1.41				
**Ownership, n (%)**										.89
	Nonprofit	3903 (27.66)		193 (26.84)		1.03				
	For-profit	10,206 (72.34)		526 (73.16)		0.99				
**Chain, n (%)**										.43
	Yes	10,627 (75.32)		551 (76.63)		0.98				
	No	3482 (24.68)		168 (23.37)		1.06				
Occupancy rate, mean (SD)			0.812 (0.2)		0.806 (0.15)		*0.03*		.46	

^a^Calculated as the probabilities of national data divided by the sample data for categorical variable or Cohen *d* (italicized) for continuous variable.

### Measures

#### Dependent Variables

Our psychometrically sound NH HIT survey has 9 subscales, from which 7 HIT maturity stages are derived [32]. The composite score has good internal consistency (Cronbach α=.86) [33]. HIT capabilities are scored 0 if the technology is “Not Available” or 1 if HIT is “Available,” as indicated by the respondents’ feedback. If an NH respondent indicates the availability of HIT capability, the respondent rates the extent of use on a scale of 1 (barely used) to 7 (extensively used). To determine the degree of integration, the respondents indicate the degree of electronic transfer of information among NH systems on a scale of 0 (not at all) to 6 (very much). The maximum range in scores for each HIT maturity dimension and domain is 0 to 100. Total HIT maturity score for all dimensions and domains combined ranges between 0 and 900. The 7 HIT maturity stages are correlated with total HIT maturity [34]. Stage 0 is the lowest stage of HIT maturity. Stage 0 indicates that HIT solutions or EHRs are nonexistent in the NH. Stage 6, the highest stage of HIT maturity, indicates the use of data by residents or residents’ representatives to generate clinical data and drive self-management.

#### Independent Variables

Five NH characteristics were included. Ownership type was collapsed into the 2 categories of “For Profit” and “Nonprofit” (nonprofit included NHs with a government classification in nursing home compare). A binary chain status variable was created. Rural-Urban Commuting Area Codes were used to classify the NHs by ZIP Codes into 4 regional locations including the following: metropolitan >50,000; micropolitan 10,000-49,999; small town 2500-9999; and rural <2500 [35]. NHs were classified into 3 classes based on the number of certified beds, including small (<60 beds), medium (60-120 beds), and large (>120 beds), which are common classifications in other NH research projects. Occupancy rate was calculated as the number of residents divided by the numbers of certified beds in the facility.

### Analysis

Probability ratios were computed to compare NH characteristics between the national data and the study sample [36]. Subsequently, weights were computed for each state. Internal consistence measured by Cronbach α and descriptive statistics of the raw and weighted total HIT maturity score as well as the 9 subscales were computed, followed by a table with stage and proportion per stage. The relationship between the NH characteristics and HIT stage was assessed. Since HIT stage is measured as a 7-point Likert scale, ordinal logistic regression was used. Unadjusted cumulative odds ratios (ORs) and associated 95% confidence intervals were computed with each independent variable entered into the regression model separately, and the adjusted cumulative ORs were computed from multivariable ordinal logistic regression where all independent variables were entered into the model. Using the continuous total HIT score as the outcome measure, multiple linear regression was performed as a sensitivity analysis to verify the significant NH factors on the HIT measure. All statistical analyses were performed using SAS version 9.3 (SAS Institute), and PROC SURVEYLOGISTIC, using variance estimators that are appropriate for survey sampled data, was used in the ordinal logistic regression analysis.

## Results

A total of 719 homes completed the survey with all 50 states and the District of Columbia being represented. The comparison of the national population of eligible NHs and the study sample is provided in Table 1, and the differences were very small. However, the sample had a larger proportion of facilities with less than 60 certified beds and a smaller proportion of larger facilities with more than 120 certified beds.

The aggregated raw HIT maturity scores are shown as Table S1 in Multimedia Appendix 1 for the 719 NHs. Median (50th percentile) scores among all 9 subscales for the 719 NHs ranged from a low of 22.22 in integration of clinical support technologies to a high of 68.97 in resident care HIT capabilities, both with a minimum and maximum score possible of 0 to 100. In 7 (78%) out of 9 subscales, at least 1 NH indicated the lowest possible score of 0, the lowest HIT maturity score indicating that HIT systems were nonexistent. Total HIT maturity scores reflect the aggregated score of all 9 subscales. At least one facility reported a total HIT maturity score of 58.32 (minimum=0), while the maximum reported by facility was 869.74 (maximum=900). The median score (440.38) closely approximated the mean score (447.2; SD 158.4), indicating a highly symmetric distribution of total HIT maturity scores. The internal consistency of the HIT maturity scores were validated (Cronbach α>.80).

**Figure 1:**
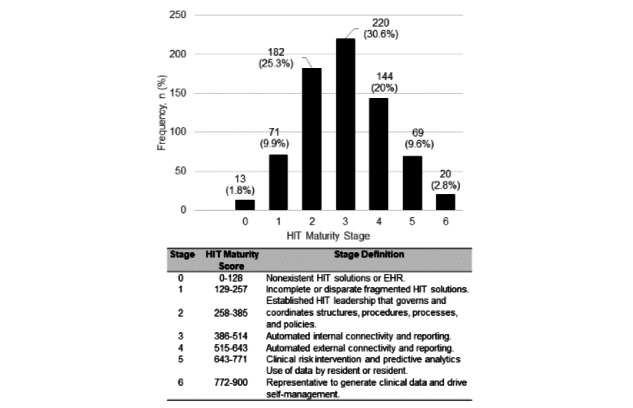
Nursing Home HIT Maturity Stages and Definitions. EHR: electronic health record; HIT: health information technology.

The weighted HIT maturity scores accounting for number of responses by state are illustrated in Table S2 in Multimedia Appendix 2. Figure 1 includes a description of HIT maturity stages and associated total HIT maturity stages in this sample. In this sample, 1.81% (13/719) of the NHs were at a stage 0, meaning that those facilities had nonexistent HIT solutions or EHRs at the time of the survey. A total of 71/719 (9.87%) facilities were at a stage 1. NHs at stage 1 have disparate or fragmented HIT systems that typically have distinct functionalities, which are not well integrated into care delivery activities. Just over 25% (n=182) of NHs have formal, established HIT leadership involved in governing and coordinating various aspects of HIT systems, putting them at stage 2. The majority of NHs surveyed (220/719, 30.6%) report achieving an HIT maturity of stage 3.

NHs at stage 3 have internal connectivity and reporting capabilities, meaning that these staff have limited capacity to communicate about care delivery with people outside their facility, such as with staff from external clinics, laboratories, or pharmacies. To be able to communicate electronically with people outside their facilities, NHs must reach a stage 4 or higher, and 32.4% (n=233) reached a stage 4 or higher. Nearly 3% (20/719 NHs) have achieved stage 6, the highest possible stage. In these facilities, data use by residents or residents’ representatives are available to generate clinical data and to drive self-management activities.

The results of the ordinal logistic regression models (Tables 2 and 3) demonstrated that the number of certified beds and location were significantly associated with HIT maturity stage, while ownership, chain status, and occupancy rate were not. The results from simple (Table 2) and multivariable (Table 3) ordinal logistic regression models are similar. From the final multivariable models (Table 3), for the number of certified beds (<60 vs >120), the odds of being at a higher stage increased by 55% for larger homes (cumulative OR=0.45; *P*=.001), when all other variables in the model were held constant. For location, the odds of being at a higher stage decreased by 55% (cumulative OR=0.45; *P*<.001) for rural homes versus metropolitan homes; the odds of being at a higher stage decreased by 43% (cumulative OR=0.57; *P*=.048) for small town homes versus metropolitan homes. The results from sensitivity analysis, using the total HIT maturity score as outcome, demonstrated similar associations between NH characteristics and HIT maturity measure (Table S3 in Multimedia Appendix 3).

**Table 2:** Simple ordinal logistic regression model assessing the relationship between nursing home characteristics and health information technology maturity stage (n=719).

Nursing home characteristics		Unadjusted cumulative odds ratio	95% CI	*P* value	
**Bed size (ref: >120)**					
	60-12	0.93	0.64 (1.35)	.71	
	<60	0.39	0.25 (0.63)	<.001^a^	
**Location (ref: metro)**					
	Micro	0.66	0.42 (1.02)	.06	
	Rural	0.36	0.23 (0.58)	<.001^a^	
	Small town	0.53	0.31 (0.91)	.02^a^	
**For-profit**					
	Nonprofit vs for-profit	0.80	0.58 (1.10)	.16	
**Chain**					
	Chain vs nonchain	1.09	0.77 (1.56)	.63	
Occupancy rate		2.21	0.83 (5.90)	.11	

^a^*P* value significant at .05 level.

**Table 3:** Multivariable ordinal logistic regression model assessing the relationship between nursing home characteristics and HIT maturity stage (n=719).

Nursing home characteristics			Adjusted cumulative odds ratio		95% CI		*P* value
**Bed size (ref: >120)**							
	60-12	0.99		0.67 (1.46)		.96	
	<60	0.45		0.28 (0.73)		.001^a^	
**Location (ref: metro)**							
	Micro	0.65		0.41 (1.03)		.07	
	Rural	0.45		0.29 (0.71)		.001^a^	
	Small town	0.57		0.33 (1.00)		.045^a^	
**For-profit**							
	Nonprofit vs for-profit	0.84		0.61 (1.17)		.30	
**Chain**							
	Chain vs nonchain	1.12		0.78 (1.61)		.54	
Occupancy rate			1.71		0.63 (4.66)		.30

^a^*P* value significant at .05 level.

## Discussion

### Principal Findings

The results from this study indicate that structural disparities in HIT maturity exist. Most facilities, nearly 68% (n=486) report being at stage 3 or lower of HIT maturity indicating they are not able to electronically communicate externally with other facilities. This lack of connectivity can result in reduced levels of electronic data sharing, leading to deficiencies in care delivery, substandard care coordination activities, and poorer resident outcomes [37]. Structural disparities in this sample appear to be most evident in HIT maturity domains and dimensions, measuring integration of clinical support technologies used for laboratory, pharmacy, and radiology services. Clinically, this makes sense, since many NHs without a hospital designation do not have these services available on site. However, that should not deter leaders from adopting systems that support higher integration and data sharing opportunities. NH leaders are challenged by a lack of financial and other incentives to adopt HIT systems that support these clinical activities and optimize care delivery [38]. Other challenges affecting outcomes of adoption supported in the literature relate to human factors design and usability issues such as excessive data entry, information overload, and slow system response times [39]. In addition to these influences, our study revealed structural disparities influenced by organizational characteristics, including NH size and location, which place some residents—usually the most vulnerable—at a disadvantage for receiving optimal care [40].

Ongoing assessments and characterization of structural disparities in NHs is crucial given 1.35 million Americans receive care in these facilities annually [41]. Without determining where disparities exist and what factors influence them, it is difficult for policy-setting organizations that oversee NH quality and care delivery to act effectively. This includes recommending and implementing strategies to reduce differences in care delivery across settings that can have a positive effect on resident outcomes. Nevertheless, leaders who focus on health care policy and disparities must be willing to promote equal opportunities across the spectrum of health care services to incentivize and enhance HIT adoption in all settings to balance these types of structural disparities to maximize resident outcomes. Otherwise, facilities such as NHs, which historically have not had the same support for promoting HIT infrastructure as other health care facilities, will certainly experience wider structural disparities and likely poorer resident outcomes.

Clearly, when incentives are provided or barriers are removed from HIT adoption, facilities will respond in ways that reduce structural disparities and promote better care delivery. To some extent, current incentive programs through meaningful use appear to be influencing HIT adoption in NHs and information-sharing practices with other clinical settings such as hospitals [8]. However, decisions to integrate electronic data sources is also dependent on organizational characteristics. For example, contrary to our findings, Burke et al [31] found that there were lower odds (OR=0.11; *P*=.04) of formal data integration between NHs and hospitals if an NH were for-profit versus not-for-profit. In a related work, Adler-Milstein et al [8] reported that higher odds (OR=1.96; *P*=.008) of sharing more complete resident information occur between Hospitals and NHs in metropolitan versus rural locations. Disparities enhanced by the size or location of a facility are likely related to resources including knowledgeable staff available to support technology implementation throughout its lifecycle. Policy makers have begun to address these deficits. For example, the Office of the National Coordinator provided funding to develop a toolkit called the Usability Change Package to support organizations that did not have ready access to usability experts and resources for EHR adoption and maintenance [42], a frequent occurrence in NHs in the United States. It is not clear how well the uptake has been or how effective these tools are for the NH industry.

### Limitations

Our survey uses broad constructs to describe structural disparities in this sample of NHs. However, we have used rigorous methods to be sure our measures have been informed by highly experienced and qualified members of the NH community [32]. One limitation, however, may be a response bias for NHs choosing not to participate. Some NH administrators may not participate because they have no technology and do not perceive relevance, which could result in an overall higher level of HIT maturity. Some NHs may not join because administrators do not have the knowledge to complete the survey. We offered help to overcome barriers by providing our contact information and answering questions as administrators participated. Our team’s increased availability and responsiveness may have reduced respondent burden, which in turn may have increased participation. Although we found some areas indicating significant differences in HIT maturity and stage when comparing some commonly used organizational characteristics, we cannot assume that lack of significance means that structural deficiencies are not present.

### Conclusion

In this national sample, we identified important structural disparities in NHs that are likely impacting the quality of care their residents are receiving. The majority of these NHs have lower HIT maturity levels, reporting a gap in connectivity with external facilities that might otherwise enhance health data sharing across different organizations. These differences could be due to inadequate infrastructures, availability of a knowledgeable workforce, or financial resources to promote higher levels of adoption. It is crucial that we begin to consistently identify a means to address these disparities, first by increasing transparency and public reporting about the trends in NH HIT maturity in the United States, followed by implementing national policies to level these deficits.

### Practice Implications

Increasingly, at the forefront of policies affecting NH care delivery is the awareness that structural disparities can place undue burden on practicing NH leaders and staff to provide high-quality care to residents. However, underneath this problem is a lack of structured and standardized means to identify and report existing structural disparities in NHs in the United States. In the absence of systematic reporting mechanisms to identify existing structural disparities in NHs, these issues will go undetected, and leaders, staff, and residents will continue to suffer the consequences.

## Appendix

Multimedia Appendix 1 Table S1.

Multimedia Appendix 2 Table S2.

Multimedia Appendix 3 Table S3.

## Abbreviations

EHR: electronic health records
HIT: health information technology
NH: nursing home
OR: odds ratio

## Data Availability

The data sets generated and analyzed during this study are not publicly available due to the project not being completed but are available from the corresponding author upon reasonable request following completion of the project.
