# Supplementary material for: Examining Structural Disparities in US Nursing Homes: National Survey of Health Information Technology Maturity
Source: JMIR Aging. 2022 Aug 23;5(3):e37482. doi: 10.2196/37482 (PMC9449826; doi:10.2196/37482)
Supplement: Multimedia Appendix 1 [file aging_v5i3e37482_app1.docx]

| **Table S1: Aggregated Raw HIT Maturity Scores by Healthcare Domains and HIT Maturity Dimensions** | | | | | | | |
| --- | --- | --- | --- | --- | --- | --- | --- |
| **Healthcare Domains** | **HIT Maturity Dimensions** | **Mean** | **Median** | **Std Dev** | **Min.** | **Max.** |  |
| **Resident Care** | **Capabilities** | 66.38 | 68.97 | 17.95 | 6.33 | 100 |  |
|  | **Extent of use** | 38.81 | 37.74 | 17.33 | 0 | 100 |  |
|  | **Integration** | 44.92 | 43.21 | 27.93 | 0 | 100 |  |
| **Clinical Support (Lab, Pharmacy, Radiology)** | **Capabilities** | 53.89 | 54.76 | 29.2 | 0 | 100 |  |
|  | **Extent of use** | 39.06 | 37.76 | 27.44 | 0 | 100 |  |
|  | **Integration** | 29.34 | 22.22 | 30.5 | 0 | 100 |  |
| **Administrative Activities** | **Capabilities** | 63 | 56.25 | 28.44 | 0 | 100 |  |
|  | **Extent of use** | 59.07 | 57.14 | 15.88 | 10 | 100 |  |
|  | **Integration** | 52.73 | 50 | 22.54 | 0 | 100 |  |
| **Total HIT Maturity** | | 447.2 | 440.38 | 158.4 | 58.3 | 869.74 |  |
| Key: Std Dev = Standard Deviation, Pctl = Percentile, Min=Minimum, Max=Maximum | | | | | | | |
