# Supplementary material for: Examining Structural Disparities in US Nursing Homes: National Survey of Health Information Technology Maturity
Source: JMIR Aging. 2022 Aug 23;5(3):e37482. doi: 10.2196/37482 (PMC9449826; doi:10.2196/37482)
Supplement: Multimedia Appendix 2 [file aging_v5i3e37482_app2.docx]

| **Table S2: Weighted HIT Maturity Summary Scores, Poststratification by State** | | | | | | | |
| --- | --- | --- | --- | --- | --- | --- | --- |
| **Healthcare Domain** | **HIT Maturity Dimensions** | **Percentile** | | **Estimate** | **Standard Error** | **95% Confidence Limits** | |
|  |  |  |  |  |  |  |  |
| **Resident Care** | **Capabilities** | **25** | **Q1** | 56.90 | 1.02 | 54.90 | 58.90 |
|  |  | **50** | **Median** | 68.83 | 0.67 | 67.52 | 70.15 |
|  |  | **75** | **Q3** | 76.55 | 0.60 | 75.37 | 77.74 |
|  | **Extent of Use** | **25** | **Q1** | 26.65 | 1.00 | 24.68 | 28.62 |
|  |  | **50** | **Median** | 37.35 | 0.69 | 36.00 | 38.71 |
|  |  | **75** | **Q3** | 50.55 | 0.80 | 48.99 | 52.11 |
|  | **Integration** | **25** | **Q1** | 22.86 | 1.44 | 20.02 | 25.69 |
|  |  | **50** | **Median** | 43.37 | 1.49 | 40.44 | 46.3 |
|  |  | **75** | **Q3** | 65.21 | 1.94 | 61.40 | 69.02 |
| **Clinical Support (Laboratory, Pharmacy, Radiology)** | **Capabilities** | **25** | **Q1** | 32.23 | 1.51 | 29.26 | 35.19 |
|  |  | **50** | **Median** | 56.46 | 2.17 | 52.21 | 60.72 |
|  |  | **75** | **Q3** | 79.60 | 1.52 | 76.62 | 82.59 |
|  | **Extent of Use** | **25** | **Q1** | 17.23 | 2.19 | 12.93 | 21.52 |
|  |  | **50** | **Median** | 39.60 | 1.68 | 36.30 | 42.89 |
|  |  | **75** | **Q3** | 61.90 | 1.42 | 59.11 | 64.70 |
|  | **Integration** | **25** | **Q1** | 0.00 | 1.97 | -3.87 | 3.87 |
|  |  | **50** | **Median** | 19.99 | 3.20 | 13.70 | 26.27 |
|  |  | **75** | **Q3** | 49.52 | 2.39 | 44.83 | 54.22 |
| **Administrative Activities** | **Capabilities** | **25** | **Q1** | 48.78 | 1.35 | 46.12 | 51.43 |
|  |  | **50** | **Median** | 57.99 | 1.67 | 54.72 | 61.26 |
|  |  | **75** | **Q3** | 91.02 | 1.67 | 87.74 | 94.30 |
|  | **Extent of Use** | **25** | **Q1** | 47.70 | 1.09 | 45.56 | 49.85 |
|  |  | **50** | **Median** | 57.70 | 0.91 | 55.92 | 59.49 |
|  |  | **75** | **Q3** | 69.93 | 0.83 | 68.30 | 71.55 |
|  | **Integration** | **25** | **Q1** | 35.68 | 1.41 | 32.90 | 38.46 |
|  |  | **50** | **Median** | 47.61 | 0.86 | 45.93 | 49.30 |
|  |  | **75** | **Q3** | 65.32 | 1.43 | 62.51 | 68.13 |
| **Total HIT Maturity** | | **25** | **Q1** | 336.60 | 7.33 | 322.21 | 351.00 |
|  |  | **50** | **Median** | 440.70 | 8.61 | 423.80 | 457.59 |
|  |  | **75** | **Q3** | 566.13 | 10.89 | 544.75 | 587.51 |
