# Supplementary material for: Examining Structural Disparities in US Nursing Homes: National Survey of Health Information Technology Maturity
Source: JMIR Aging. 2022 Aug 23;5(3):e37482. doi: 10.2196/37482 (PMC9449826; doi:10.2196/37482)
Supplement: Multimedia Appendix 3 [file aging_v5i3e37482_app3.docx]

| **Table S3: Multivariable linear regression model assessing the relationship between nursing home characteristics and HIT maturity score (n=719)** | | | | | |
| --- | --- | --- | --- | --- | --- |
| Nursing home characteristics | | B | 95% Confidence Interval | | P value |
| Bedsize (ref: >120) | 60-12 | -5.88 | -41.25 | 29.50 | 0.744 |
|  | <60 | -69.37 | -112.87 | -25.87 | 0.0018* |
| Location (ref: Metro) | Micro | -42.24 | -83.44 | -1.03 | 0.0445* |
|  | Rural | -75.14 | -110.44 | -39.84 | <.0001* |
|  | Small Town | -42.75 | -95.32 | 9.82 | 0.098 |
| For Profit | Non-profit vs profit | -9.64 | 38.52 | 19.24 | 0.513 |
| Chain | Chain vs non-Chain | 20.02 | -12.08 | 52.12 | 0.221 |
| Occupancy Rate |  | 65.52 | -26.38 | 157.42 | 0.162 |
| *: P value significant at 0.05 level. | | | | | |
